# Supplementary material for: Nonvisual Support for Understanding and Reasoning about Data Structures
Source: Proc SIGCHI Conf Hum Factor Comput Syst. Author manuscript; Available in PMC 2026 Jul 17. (PMC13374576; doi:10.1145/3772318.3791656)
Supplement: Supplemental Materials [file NIHMS2178337-supplement-Supplemental_Materials.zip › UserStudy.docx]

## Tasks Questions

**T1. Element Location**

1. Is the element ‘54’ in the array? If so, what position/index is it at?
2. What element comes immediately before ‘63’?
3. How many numbers in the array are greater than or equal to 10?

*TX. Order/Sorting*

Is this array sorted in increasing order?

**T2. Parent/Child Identification**

What is the value of the root node’s right child?

**T3. Leaf Node Identification**

How many leaf nodes are in the tree?

**T4. BST Property Check**

Do all the nodes fit the BST property?

**T5. Binary Search**

1. Does this tree contain the number 0?
2. Does this tree contain the number 6?
3. Does this tree contain the number 9?

## Semi-Structured Interview Questions

- Was it easy or difficult to find the information you needed within each representation?
- Of the three representations (table, keyboard-friendly, tactile), which one did you find most helpful for understanding the structures? Why?
- How important was it for you to have more than one way to access the same information?
  - *Did switching between formats help your understanding, or did you mostly stick with one?*
- Was there a representation that you found less helpful or more difficult to use?
  - *What made it harder to work with?*
- Did the representations make the structure of the data clear to you overall?
  - *If not, what would have made it clearer?*
- How did what you learned about arrays impact your understanding of binary trees?
- Is there anything else you’d like to share about your experience today?
