# Supplementary material for: Nonvisual Support for Understanding and Reasoning about Data Structures
Source: Proc SIGCHI Conf Hum Factor Comput Syst. Author manuscript; Available in PMC 2026 Jul 17. (PMC13374576; doi:10.1145/3772318.3791656)
Supplement: Supplemental Materials [file NIHMS2178337-supplement-Supplemental_Materials.zip › Codebook.docx]

Codebook Overview

The following codebook documents the 78 focused codes that resulted from our analysis. We began with 444 open codes capturing fine-grained aspects of participant feedback. Through iterative comparison and team discussion, these were consolidated into 78 focused codes, which were then grouped into 17 categories and organized under 5 overarching themes. For each code, we provide a short definition.

# Table of Contents

[Theme: Representation Affordances & Limitations](#_m6thd7sw8ck6)

[Tactile Affordances](#_x0j0835a4kd2)

[Navigable Affordances](#_tcatbthlloq8)

[Tabular Affordances](#_furiapr4yl8c)

[Tactile Navigation Patterns](#_rzbvpu2cx9vv)

[Digital Navigation Approaches](#_x6zxkp4aoy17)

[Navigable Limitations](#_gifpmu37cbey)

[Theme: Multimodal Integration & Strategic Use](#_rh4mxsyrvvu9)

[Strategic Integration](#_nc9491hkr)

[Synergistic Effects](#_c31fjhjok4yy)

[Context-Dependent Use](#_4e23qzy8ouqd)

[Theme: Learning Processes & Mental Model Development](#_i996mwcax3hm)

[Mental Model Construction](#_exzbblzd5o6m)

[Learning Barriers & Misconceptions](#_y9ixulenbj8m)

[Learning Strategies](#_761ixe8nantv)

[Theme: Conceptual Understanding & Algorithm Application](#_lam0fobt6aao)

[Prior CS Experience Effects on Understanding](#_x6he5ny2s6pt)

[Semantics](#_mg29dqo20o5t)

[Structural Understanding](#_ij1ky6kxh8xd)

[Algorithmic Reasoning](#_543egydm469n)

[Theme: Accessibility Design Implications](#_wgo9s6z37dbh)

[Educational Implications](#_myhej9ktsz1f)

#

Full Codebook Entries

# Theme #1: Representation Affordances & Limitations

Participants evaluated how different representations (tactile, navigable/digital, and tabular) supported or hindered their understanding. They highlighted unique strengths of each format while also identifying their limitations.

## Tactile Affordances

**Tactile Intuitiveness:** When participants described understanding concepts instinctively through touch, without requiring extra explanation.

**Tactile Comprehension Advantages:** Specific benefits participants reported when using touch to understand information (e.g., clarity, depth, or precision of understanding).

**Ease of Tactile Navigation:** When tactile graphics allowed participants to quickly move to the information they needed or advance to the next step without confusion.

**Tactile Supports Mental Visualization:** Instances where tactile graphics helped participants form or strengthen mental images of the structures.

**Tactile-Supported BST Understanding:** Ways in which tactile representations specifically improved participants’ comprehension of binary search trees.

**Strong Tactile Preference:** Explicit statements of marked preference for tactile representations compared to other modalities.

**Tactile Efficiency Trade-Offs:** Reflections on both the benefits and the costs of tactile representations, such as requiring more time or effort while offering other advantages.

**Positive Attitudes Toward Braille:** Favorable views participants expressed about Braille as a tactile medium for accessing the information.

**Tactile Perception Challenges:** Difficulties participants faced in perceiving or interpreting tactile information.

**Braille Format Challenges:** Specific obstacles related to how Braille should be formatted for these structures, including mentions of standards or conventions.

## Navigable Affordances

**Navigable Tree Supports Efficient Hierarchical Access**: When participants described tree-based representations as enabling structured, top-down navigation and helping them understand screen reader levels.

**Digital Efficiency Trade-Offs**: Reflections on the benefits and drawbacks of digital representations—easy to access for most, but sometimes adding cognitive or interaction overhead.

**Navigable Tree/List Has Familiar Interaction Patterns:** When participants recognized common screen reader navigation styles (e.g., expand/collapse trees, list navigation with arrow keys).

**Digital Design Awareness:** Participant comments on how the design of digital representations (layout, interface elements, headings) supported familiarity and comprehension.

**Missing Physical/Spatial Aspects:** When participants noted what was lost in digital formats compared to tactile ones, such as spatial layout, depth, or embodied sense of structure.

**Verbosity and Constraints of Current Digital Standards:** Participant frustrations with limitations of existing standards (e.g., ARIA, screen reader behaviors) that produced excessive verbosity or restricted flexibility.

**Digital Representation Limitations:** General challenges participants identified with digital formats, such as incomplete support for comprehension or lack of flexibility.

**List Provides Bidirectional Linear Access:** When participants noted that list formats supported clear forward and backward navigation but only in a linear sequence.

## Tabular Affordances

**Table Works Well for Arrays:** When participants described tables as effective for representing arrays.

**Table Provides Systematic Organization:** When participants noted that tables offered clear, structured organization of information.

**Context-Dependent Table Utility:** Reflections that the usefulness of tables depended on the specific task or context.

**Table Familiarity and General Preference:** When participants expressed comfort with tables or a general preference for them as a format.

**Table Enables Flexible Access Patterns:** When participants highlighted that tables supported multiple ways of navigating or interpreting the tabular information.

**Table Structural Inadequacy for Trees:** When participants identified that tables did not capture hierarchical structures effectively.

**Table Undermines Learning Objectives:** When participants felt that relying on tables reduced deeper learning or understanding of the underlying concepts.

## Tactile Navigation Patterns

**Edge-Following Strategies:** Instances where participants used connecting lines or edges in tactile diagrams to guide their navigation.

**Tactile Value & Index Location:** When participants searched for or referenced specific values or indices within tactile representations.

**Free Exploration vs. Strategic Exploration:** Descriptions of participants either freely scanning diagrams or using deliberate, goal-directed navigation strategies.

## Digital Navigation Approaches

**Screen Reader Navigation Workarounds & Barriers:** When participants described strategies for overcoming, or challenges posed by, screen reader navigation constraints.

**Sequential vs. Strategic Traversal Approaches:** Reflections on moving step by step through information versus skipping ahead or targeting specific elements.

**Learning & Guidance in Digital Navigation:** Instances where participants highlighted the need for practice, instruction, or cues to effectively navigate digital formats.

**Tree Navigation Confusion:** When participants expressed difficulty understanding or keeping track of navigation within digital tree structures.

**Table & Column-Based Navigation Strategies:** Descriptions of participants using table rows or columns as a systematic way to move through or find specific information.

**List Navigation Simplicity:** When participants noted that list formats offered straightforward, easy-to-use navigation.

**Navigation Preferences:** Explicit statements of which navigation style or format participants preferred.

## Navigable Limitations

**Navigation Confusion and Difficulty Locating Nodes:** Instances where participants struggled to find specific nodes while navigating.

**Command Complexity in Digital Tree:** When participants described the number or difficulty of commands needed to navigate digital tree structures.

**Navigable Tree Needs Interaction Improvements:** Participant suggestions that tree navigation could be improved with clearer or simpler interaction methods.

**Confusion and Frustration with Expand/Collapse Interaction:** When participants reported difficulty or annoyance with expand/collapse features in navigable trees.

**Mismatch Between Expected and Actual Navigation Model:** Instances where participants’ expectations of how navigation should work differed from how it actually functioned.

# Theme #2: Multimodal Integration & Strategic Use

Participants combined representations to verify, switch, and adapt strategies depending on task, data structure, and complexity. They saw clear benefits in having multiple options available.

## Strategic Integration

**Verification Across Modalities:** When participants checked or confirmed information by comparing across tactile, digital, or table representations.

**Strategic Switching:** Instances where participants intentionally switched between representations to solve a task more effectively.

**Data Structure-Specific Switching Effects:** When participants switched representations in ways that depended on the type of data structure (e.g., arrays vs. trees).

**Complexity-Driven Preferences:** When participants’ mentioned their choice of representation was influenced by the complexity of the task.

## Synergistic Effects

**Value of Multiple Options:** Participant reflections on the benefits of having more than one representation available.

**Value of Combining Modalities:** When participants highlighted advantages of using representations together rather than separately.

**Multi-Representation Comfort:** Expressions of confidence or ease when multiple representations were available.

## Context-Dependent Use

**Task-Specific Utility:** When participants noted that certain representations were more useful for particular tasks.

**Context-Dependent Preferences:** Instances where participants expressed that their representation preference depended on the situation or context.

**Systematic Representation Rankings:** When participants explicitly ranked representations in order of usefulness or preference.

# Theme #3: Learning Processes & Mental Model Development

Participants reflected on how representations supported or hindered learning, mental model building, and strategy use.

## Mental Model Construction

**Multimodal Mental Model Building:** When participants described constructing mental models by drawing on multiple representations together.

**Tactile Supports Mental Model Construction:** Instances where tactile graphics specifically helped participants form or reinforce mental models.

**Instructional Sequencing Effects:** When participants noted that the order in which representations were introduced affected their understanding.

**Table Provides Limited Mental Model Support:** When participants felt that tables offered only partial or insufficient support for building deeper mental models.

## Learning Barriers & Misconceptions

**Terminology Barriers:** Difficulties participants expressed due to unfamiliar or confusing terms used in instruction or representations.

**Memory Management Challenges:** When participants struggled to manage remembering information while working through tasks.

**Representation Mapping Difficulties:** Instances where participants had trouble connecting one representation to another or aligning them conceptually.

**Perceptions Around Cheating and Shortcuts:** Participant reflections on whether using certain representations felt like bypassing learning goals or taking shortcuts.

## Learning Strategies

**Learning Through Analogy/Metaphor:** When participants used comparisons or metaphors to make sense of data structures.

**Metacognitive Learning Strategies:** Instances where participants described monitoring, reflecting on, or adjusting their own learning process.

**Working Memory Preferences:** When participants expressed preferences shaped by their ability to hold and manipulate information in working memory.

**Transfer Across Data Structures:** When participants described applying knowledge or strategies learned with one data structure to another.

# Theme #4: Conceptual Understanding & Algorithm Application

Representations shaped how participants understood structural roles, spatial organization, and algorithmic processes, often mediated by their prior CS experience.

## Prior CS Experience Effects on Understanding

**Learning Curve & Familiarity:** When participants noted that prior exposure or practice shaped how quickly they understood a representation.

**Past Experience Effects:** Instances where participants’ previous coursework or coding background directly influenced their comprehension.

**Progressive Understanding:** When participants described gradually improving their grasp of concepts over the session or with the assumption it would with repeated exposure.

## Semantics

**Semantic Role Comprehension:** Instances where participants correctly identified and understood the functional roles of elements within a structure.

**Semantic Interpretation Challenges:** When participants struggled to interpret the roles or relationships between elements in a structure.

**Role Identification in Structures:** When participants explicitly identified specific roles (e.g., parent, child, index) within a representation.

## Structural Understanding

**Spatial Positioning:** When participants described the importance of spatial placement of elements for understanding the structure.

**Layout & Orientation:** Instances where participants commented on the overall arrangement or orientation of a representation.

**Tactile Layout Comprehension:** When participants described how tactile layouts specifically supported or hindered their understanding.

## Algorithmic Reasoning

**Algorithm Application and Verification:** When participants applied algorithms to structures or checked their correctness through a representation.

**Representation-Specific Algorithm Support:** Instances where participants found a representation particularly useful for carrying out or reasoning through an algorithm.

**Reasoning Strategies and Verbalization:** When participants explained their thought process or reasoning aloud while working through a task.

# Theme #5: Accessibility Design Implications

Participants emphasized the importance of accessible instructional design and noted that accessibility features can benefit a broad range of learners, not just those with vision impairments.

## Educational Implications

**Value of Accessible Instruction:** Participant reflections on the importance of accessible materials in classrooms.

**Educational Accessibility Barriers:** When participants identified obstacles in current teaching practices that limit accessibility.

**Accessibility Benefits Outside BVI:** Reflections that accessible approaches designed for blind or low-vision learners also benefit broader disability communities.
