# Supplementary figures and images for: Nonvisual Support for Understanding and Reasoning about Data Structures
Source: Proc SIGCHI Conf Hum Factor Comput Syst. Author manuscript; Available in PMC 2026 Jul 17. (PMC13374576; doi:10.1145/3772318.3791656)

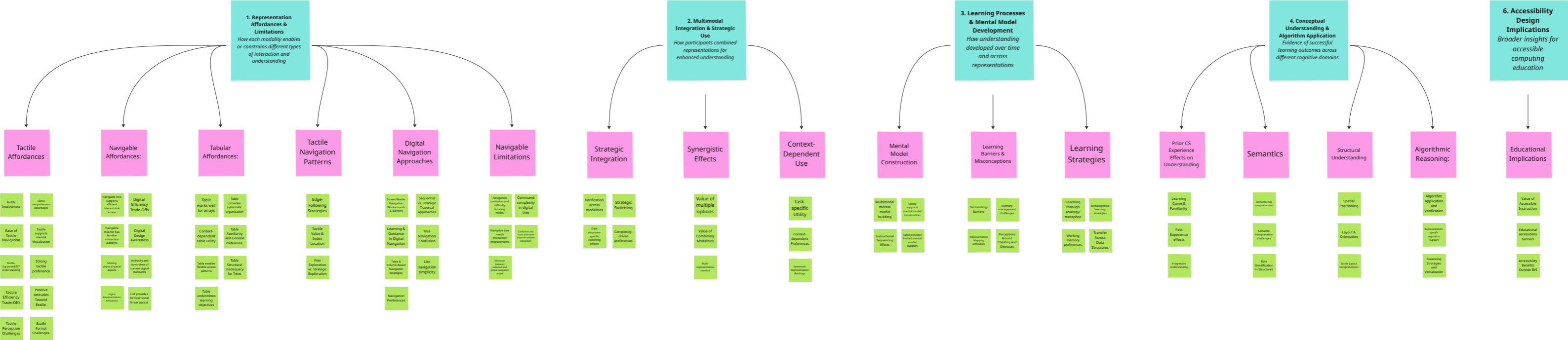

Supplement: Supplemental Materials [file NIHMS2178337-supplement-Supplemental_Materials.zip › CodeMapping.pdf]
